# Gene therapy in animal models of autosomal dominant retinitis pigmentosa

**Published:** 2012-10-06

**Authors:** Brian Rossmiller, Haoyu Mao, Alfred S. Lewin

**Affiliations:** Department of Molecular Genetics and Microbiology, University of Florida, Gainesville, FL

## Abstract

Gene therapy for dominantly inherited genetic disease is more difficult than gene-based therapy for recessive disorders, which can be treated with gene supplementation. Treatment of dominant disease may require gene supplementation partnered with suppression of the expression of the mutant gene either at the DNA level, by gene repair, or at the RNA level by RNA interference or transcriptional repression. In this review, we examine some of the gene delivery approaches used to treat animal models of autosomal dominant retinitis pigmentosa, focusing on those models associated with mutations in the gene for rhodopsin. We conclude that combinatorial approaches have the greatest promise for success.

## Introduction

Vision is one of our most valuable senses, allowing for the detection of a single photon at night and high acuity perception in the day. Thus, retinal degenerative diseases can have a large impact on the quality of life. One such disease, retinitis pigmentosa (RP), is responsible for vision loss in 1 in 4,000 people worldwide [1]. As retinitis pigmentosa is initially a disease of the rod photoreceptors, vision loss is first perceived in the periphery and at night. In retinal images, dark pigmentary deposits termed “bone spicules” are observed [2,3]. As the rods continue to die, a paling of the optic nerve, spreading of pigmentary deposits, thinning of retinal vessels, and decrease in electroretinogram (ERG) response are observed [2,3]. Only after the loss of rods do the cones of the macula begin to die, causing near total blindness in afflicted individuals [2,3].

Retinitis pigmentosa is transmitted in autosomal dominant, autosomal recessive, sex-linked dominant, and sex-linked recessive modes of inheritance [3]. More than 30 genes and many different mutations, over 100 mutations in rhodopsin alone, have been associated with retinitis pigmentosa [4,5]. This genetic heterogeneity is associated with differences in rate and the extent of the degeneration. Accounting for 30%–40% of all cases of retinitis pigmentosa, autosomal dominant retinitis pigmentosa (ADRP) is the most common mode of inheritance and is the consequence of mutations in 24 known genes (Table 1) [6].

**Table 1:** Genes and loci associated with ADRP.

**Protein**	**Gene**	**Location**
Bestrophin-1	*BEST1*	11q13
Carbonic anhydrase IV	*CA4, RP17*	17q23.2
Cone-Rod Homeobox	*CRX*	19q13.32
Fascin homolog 2	*FSCN2, RP30*	17q25.3
Guanylate cyclase activator 1B	*GUCA1B, RP48*	6q21.1
Inosine monophosphate dehydrogenase 1	*IMPDH1, RP10*	7q32.1
kelch-like protein 7	*KLHL7, RP42*	7p15.3
Nuclear receptor subfamily 2 group E member 3	*NR2E3*	
Neural retina leucine zipper	*NRL, RP27*	14q11.2
OSBP-related protein 1	*ORP1, DCDC4A, RP1*	8q12.1
pre-mRNA processing factor 3	*PRPF3, RP18*	1q21.2
pre-mRNA processing factor 31 homolog	*PRPF31*	19q13.342
pre-mRNA processing factor 6	*PRPF6,rp60*	20q13.33
pre-mRNA processing factor 8	*PRPF8*	17.13.3
Peripherin 2	*PRPH2, RDS, RP7*	6q21.1
Rhodopsin	*RHO*	3q22.1
Retinal outer segment membrane protein 1	*ROM1*	11q12.3
Retinitis pigmentosa 1 protein	*RP1, L1*	8q23.1
Unknown	*RP63*	6q23
Retinitis pigmentosa 9 protein	*RP9*	7p14.3
Retinal pigment epithelium-specific protein	*RPE65, RP20*	1p31.2
Semiphorin	*SEMA4A, RP35*	1q22
Proto-oncogene tyrosine-protein kinase MER	*MERTK, RP33*	2q11.2
Topoisomerase I-binding arginine/serine-rich protein	*TOPORS*	9q21.1

Known gene and loci locations for ADRP causing mutations and associated proteins or loci names. References are at RetNet.

Currently, there are no effective treatments for ADRP. Nutritional therapy featuring vitamin A or vitamin A plus docosahexaenoic acid reduces the rate of degeneration in some patients [7]. Retinal analogs and pharmaceuticals functioning as chaperones show some progress in protecting the retina in animal models [8-11], and several antioxidant studies have shown lipophilic antioxidant taurousodeoxycholic acid (TUDCA), metallocomplex zinc desferrioxamine, N-acetyl-cysteine, and a mixture of antioxidants slow retinal degeneration in rodent rd1, rd10, and Q344ter models [12-15]. Although TUDCA is in clinical trials for other indications, it has not been tested in patients with retinal disease. A clinical trial is under way to test the efficacy of the protein deacetylase inhibitor valproic acid as a treatment for retinitis pigmentosa (Clinicaltrials). Valproic acid blocks T-type calcium channels and voltage-gated sodium channels [16], and is associated with significant side effects such as hearing loss and diarrhea. Therefore, the use of valproic acid as a treatment for retinitis pigmentosa has been questioned [17,18].

### Rhodopsin mutations

**Figure 1:**
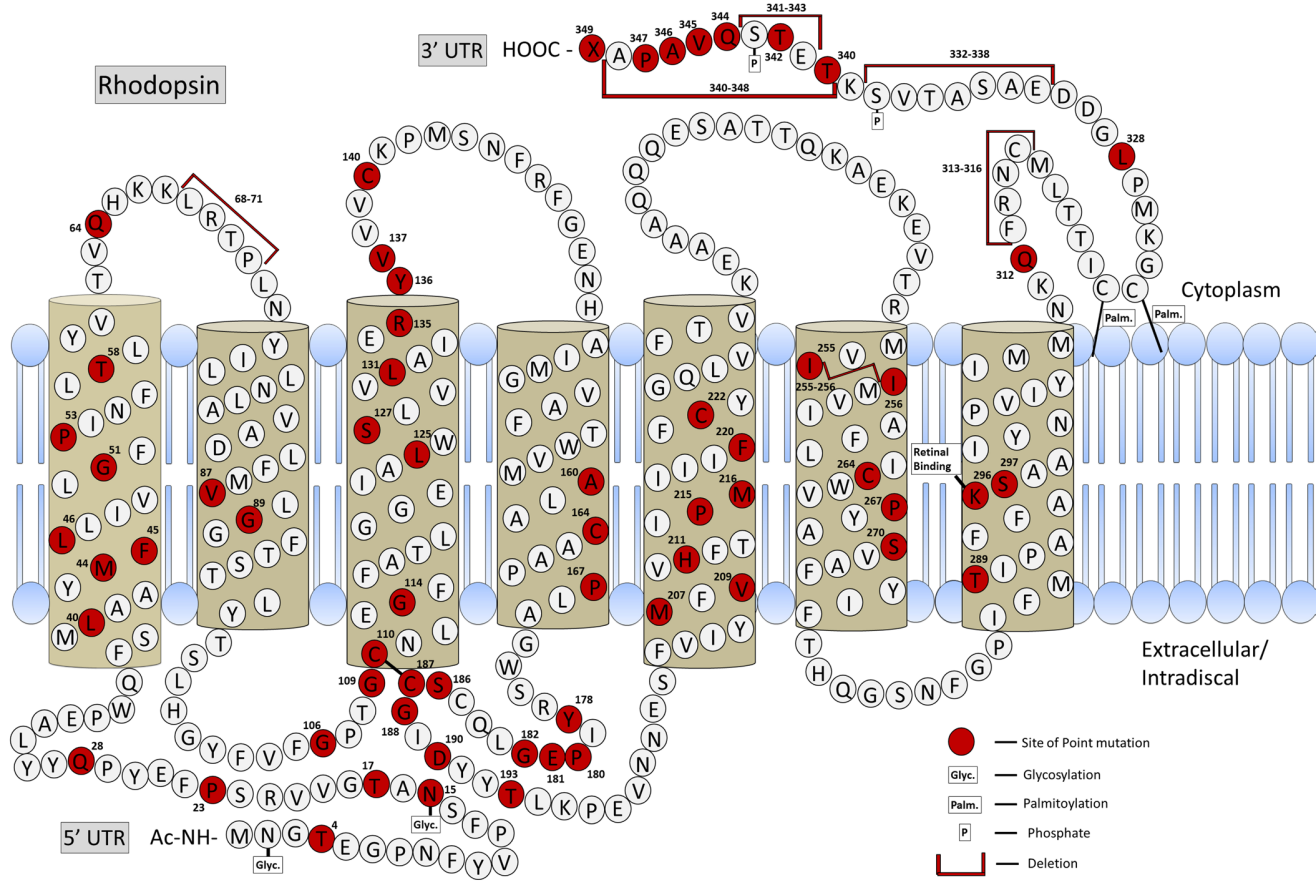
Human rhodopsin illustrating sites of known mutations or deletions. This figure is based on an illustration at RetNet.

Despite the range of genes responsible for ADRP, approximately 30% of ADRP arises from mutations in the rhodopsin gene [19], and therefore, we focus our attention on treatment of mutations affecting the rhodopsin gene (*RHO* in humans and *Rho* in mice). Numerous alterations in *RHO* cause ADRP (Figure 1; RetNet). These mutations do not localize to any specific regions of the protein, suggesting that functional and stable rhodopsin tolerates few amino acid changes. In fact, human rhodopsin protein differs at only 13 positions from the rabbit, 17 positions from the cat, and 18 positions from the mouse. The maximum sequence identity is ≥95% among all of these organisms. The consequences of particular mutations have been analyzed in transfected cells and animal models, sometimes with conflicting results [20]. Rods are also highly susceptible to changes in rhodopsin expression and translocation to the outer segment of photoreceptors, as rhodopsin composes greater than 90% of the outer segment protein [1,5,21-23].

ADRP mutations in rhodopsin have been placed into categories based on the mutations’ impact on protein folding and trafficking. Class I mutations result in normal rhodopsin folding, but the protein is not efficiently transported to the outer segment and has constitutive activation or an increased transducin activation rate [24]. Mutations affecting the C-terminus of rhodopsin, such as P347S, fall into this class. Class II mutations result in opsin that folds improperly, is retained in the endoplasmic reticulum (ER), and does not reconstitute with the 11-cis-retinal chromophore [24,25]. Rhodopsin folding and function can be affected by alterations in post-translation modification sites for glycosylation and disulfide bond formation [21,26]. Mutations affecting glycosylation at N2 and N15 and the disulfide bond between C110 and C187 are known causes of ADRP [26-28]. The T17M mutation results in rapid degeneration especially evident in the inferior retina. In some cases, degeneration from this class can be slowed with restricted light exposure [29,30]. The most common class II mutation in *RHO* in North America is P23H. P23H has been shown to be a target of the endoplasmic reticulum associated protein degradation (ERAD) effector valosin containing protein (VCP), a chaperone responsible for removing misfolded proteins from the ER for proteasomal degradation. In a rat model of ADRP, P23H rhodopsin stimulates the unfolded protein response [31,32]. Other mutations in *RHO* have been classified based on the stability of the protein or by the constitutive activation of the visual transduction pathway [24]. G90D, for example, leads to constitutive activation of transducin and causes congenital stationary night blindness.

Although ADRP mutations affect rod photoreceptors directly, they ultimately result in the death of cone photoreceptors and the loss of central vision. The mechanism by which *RHO* mutations cause cone cell death is not known, but several mechanisms have been proposed. These include (i) release of endotoxins, (ii) loss of rod trophic factors, (iii) loss of contact with the retinal pigment epithelium (RPE), (iv) activation of Müller glial cells, (v) increased oxygen toxicity, and/or increased metabolic load [5,33].

### Animal models of autosomal dominant retinitis pigmentosa

The need for an effective therapy for ADRP has led to the creation of several mammalian models, and the use of these models has aided in the ongoing development of treatments focusing on neurotrophic factors, gene suppression, and gene replacement using viral or non-viral delivery. Due to the high degree of sequence homology in rhodopsin, mutations affecting human families can often be studied in animals. Because of their genetic malleability, mice and rats provide the most common models with rhodopsin mutations, including P23H, T17M, P347S, and S334X, present on transgenes, leading to retinal degeneration at varying rates. Chemical mutagenesis has led to the isolation of mouse lines bearing other mutations at the *Rho* locus, and Sakami et al. have produced a line bearing P23H *Rho* knocked into the endogenous locus [34,35]. The canine model of ADRP has provided insight into the risks of clinical illumination and the benefits of neurotrophic factors as potential treatments [36-39]. Porcine models of ADRP are also available. The size of the pig eye has lent it to testing of surgical treatments for ADRP such as retinal sheet transplants [40,41], but the large size of domestic swine make them difficult to work with as adults. Ross et al. have recently described an inbred line of miniature pigs bearing a human P23H transgene, and these may prove to be a tractable model [42].

### Preclinical outcome measurements

Assessing the course of retinal degeneration and the success of treatment in rodent models of retinal disease employs some of the same technologies used to evaluate patients with retinitis pigmentosa: ERG, spectral domain optical coherence tomography (SD-OCT), and digital fundus imaging. In addition, light and electron microscopy on fixed tissue can be used to measure histopathology in animal models of ADRP.

Full-field ERG is typically used to assess retinal function in mouse and rat models of ADRP [22,43-46]. Because the retinas of these nocturnal rodents are rod-rich, dark-adapted (scotopic) ERG measurements are recorded, though light-adapted (photopic) ERG amplitudes may be affected in models of cone-rod dystrophy or late in the course of RP models. Results are typically presented as a-wave and b-wave maximum amplitudes as a function of flash intensity or as a-wave and b-wave amplitudes as a function of time at a single flash intensity. Because the full-field ERG recordings measure the response of the whole retina, local improvements in retinal function resulting from localized gene delivery may be difficult to accurately measure. Because ERG responses from both eyes are typically recorded simultaneously, a sham-treated contralateral eye can be used as the control for eyes treated with gene therapy.

Visual function can also be evaluated with behavioral analysis [47]. Although the Morris water maze can be used in dim light to measure the recovery of rod function after gene therapy [48], optokinetic (OptoMotry ™ CerebralMechanics, Lethbridge, AB, Canada) analysis has been used to measure visual acuity and contrast sensitivity in mice following gene therapy or cell transplantation therapy for retinal degeneration [49-51]. Since optokinetic measurements are performed in a lit room, they are primarily useful for measuring photopic function.

SD-OCT instruments have become widely used for assessing retinal degeneration in animal models in the past few years [49,52-55]. OCT is an interferometer-based imaging technology providing cross-sectional images of tissues transparent to infrared illumination. Instruments from Heidelberg Engineering or Bioptigen permit measurement of retinal thickness and of the thickness of the outer nuclear layer (ONL), which is often used to assess the survival of photoreceptor cells. The ONL is measured between highly reflective bands corresponding to the outer plexiform layer and the external limiting layer of the retina. SD-OCT permits the same cohort of animals to be followed longitudinally over the course of retinal degeneration and response to treatment. An added value of SD-OCT for preclinical gene therapy testing is that retinal detachment resulting from subretinal injections is readily detected, allowing these animals to be removed from the analysis.

Fundus imaging using film, and, more recently, digital imaging, has long been used to qualitatively monitor the course of retinal degeneration in patients and in research animals [56], and many of the mouse models of retinal degeneration were first recognized by fundus phenotype [57]. Some instruments used for SD-OCT are also capable of digital fundus imaging, and this is useful because both image modalities can be used in the same session of anesthesia. Unlike RP in humans, which is recognized by pigment deposition, mouse models of retinal degeneration are characterized by hypopigmented regions or spots. These regions may become larger during the course of retinal degeneration, and this progression may be arrested with successful gene therapy [58].

Although SD-OCT provides a measurement of ONL thickness, SD-OCT does so only in the central 30% of the retina, so that light microscopy is necessary to assess retinal structure in the periphery. In addition, the photoreceptor outer and inner segments are not well resolved by SD-OCT, so that the structure and thickness of these layers must be measured by microscopy and morphometry. Typically, eyes are fixed in glutaraldehyde and paraformaldehyde, dehydrated, and then embedded in plastic or in paraffin. Sections are made along the vertical meridian through the optic nerve head, and the thickness of the retinal layers is measured at regular distances from the optic nerve head in the superior and the inferior retina [59].

### Therapeutic approach: neurotrophic factors

Neurotrophic factors (NTFs) play a large role in the development and maintenance central nervous system including the retina. One RPE cell, for instance, contacts 40 cones and rods and secretes NTFs both to the choroid and the photoreceptors [6]. Rods also produce a neurotrophic factor, rod derived cone viability factor (RdCVF), a thioredoxin-like protein lacking the oxoreductase activity, that influences cone survival [60,61]. The injection of RdCVF protein in P23H rats increased cone density 19% [62]. RdCVF also increased the survival of retinal explants from *rd1* mice, an effect that could be blocked if RdCVF was immunodepleted [60]. Basic fibroblast growth factor (bFGF) comprises 22 types, but only bFGF2 has been shown to preserve photoreceptors in RCS rats for approximately two months [59]. Application of acidic fibroblast growth factor (aFGF) and bFGF in the subretinal space and the vitreous preserved ONL thickness in RCS rats with diminished RPE engulfment of photoreceptor outer segments [59]. Brain derived neurotrophic factor (BDNF) is expressed in many locations, including the brain, motor neurons, and the retina. When administered with other NTFs, including ciliary-derived neurotrophic factor (CNTF) and glial-derived neurotrophic factor (GDNF), BDNF increased photoreceptor preservation but caused the cells to revert to a more primitive state and reduced synthesis of rhodopsin [63,64]. Pigment epithelium derived factor (PEDF) is being studied for treatment of age-related macular degeneration and is known to be secreted by Müller glial cells and the RPE [65,66]. PEDF protects cells from glutamate toxicity and photoreceptors from excessive light. PEDF also slows degeneration in *rd1* mice. Two of the more promising NTFs, CNTF, and GDNF, have been delivered by gene transfer. These factors could promote cell survival in different forms of ADRP and thus prolong useful vision and the window for therapeutic treatment.

### Ciliary-derived neurotrophic factor

CNTF is a member of the interleukin-6 (IL-6) family of cytokines [67]. Its effectiveness is limited by a short half-life of 120–400 min, which rules out bolus injections [68]. Research has instead focused on a virally delivered CNTF gene or on mammalian cell lines encapsulated in an implantable device, NT-501 [67,68]. Mice transgenic for a dominant negative mutant (P216L) of rds/peripherin (*Prph2*) showed photoreceptor protection, but reduced ERG and decreased photoreceptor gene expression when injected with an adeno-associated virus (AAV) expressing a secreted form of CNTF in the subretinal space [69]. Loss of retinal function in *Prph2* mutant mice was also reported by Schlichtenbrede et al. [70]. CNTF delivered as a protein was beneficial in maintaining ONL thickness in rats bearing the S334X mutation of rhodopsin [71]. CNTF was even shown to cause regeneration of cone outer segments (OS). Liang and colleagues, also working with rats transgenic for S334X or P23H *RHO* and *Prph2^rd2/rd2^* mice, had similar results, showing a preservation of retinal histology months after the photoreceptors began to degenerate [72]. A reduction in the ERG a-wave and b-wave responses and an increase in Müller glial cell activation were also noted. Similar results were also seen, using NT-501 during human phase I clinical trials [67].

### Glial-derived neurotrophic factor

The benefits of GDNF, a member of the transforming growth factor-beta family, to the central and peripheral nervous system have been studied for years: GDNF prolongs dopaminergic neuron and dorsal root ganglion cell survival. GDNF and its receptors are naturally expressed in the retina [40]. AAV expression of GDNF, driven by a CMV enhancer/chicken beta-actin (CBA) promoter, slowed photoreceptor degeneration and maintained ONL thickness and ERG response in rats bearing a transgene containing an ADRP *RHO* mutation (S334X) [73]. There are several possible mechanisms for the protective effects of GDNF. First, GDNF may elicit effects through increases in the levels of other NTFs, including BDNF and fibroblast growth factor-2 [74,75]. Second, GDNF could protect the metabolically active retina from reactive oxygen species, already shown in kainate injected pyramidal neurons [76]. Since GDNF receptors are expressed on Müller glial cells but not in photoreceptors, GDNF probably protects photoreceptors indirectly, acting through Müller cells [40].

Retinas of rats treated with AAV-GDNF had no indication of inflammatory cell infiltration, no decrease in retinal ganglion cells, and no statistical effect on ERG response after 1 year of expression [77]. GDNF not only increased retinal cell survival in vivo but also enhanced the survival of axotomized retinal ganglion cells and light response of transplanted retinal sheets [78,79]. Using the *Prph2^rd2/rd2^* mouse model and the RCS rat model of retinal degeneration, Buch et al. showed that AAV-CBA-GDNF led to significant functional improvement based on ERG amplitudes and preservation of the ONL especially when combined with gene supplementation [80].

### Neurotrophic factor disadvantages

Neurotrophic factors have the disadvantage of non-specific action. CNTF in the vitreous has demonstrated effects on retinal ganglion cells, Müller glial cells, astrocytes, and cone and rod photoreceptor cells [81-83]. CNTF has been shown to cause regeneration of cone outer segments but at the expense of rod function [83]. The knockdown of photoreceptor gene expression by CNTF is transient, and normal gene expression is detected when CNTF is no longer present. GDNF is also known to affect various cell types and possibly dose-dependent toxicity [84]. Application of neurotrophic factors as proteins could allow degeneration to be slowed until a long-term treatment has been administered.

### Therapeutic approach: gene suppression and replacement

**Figure 2:**
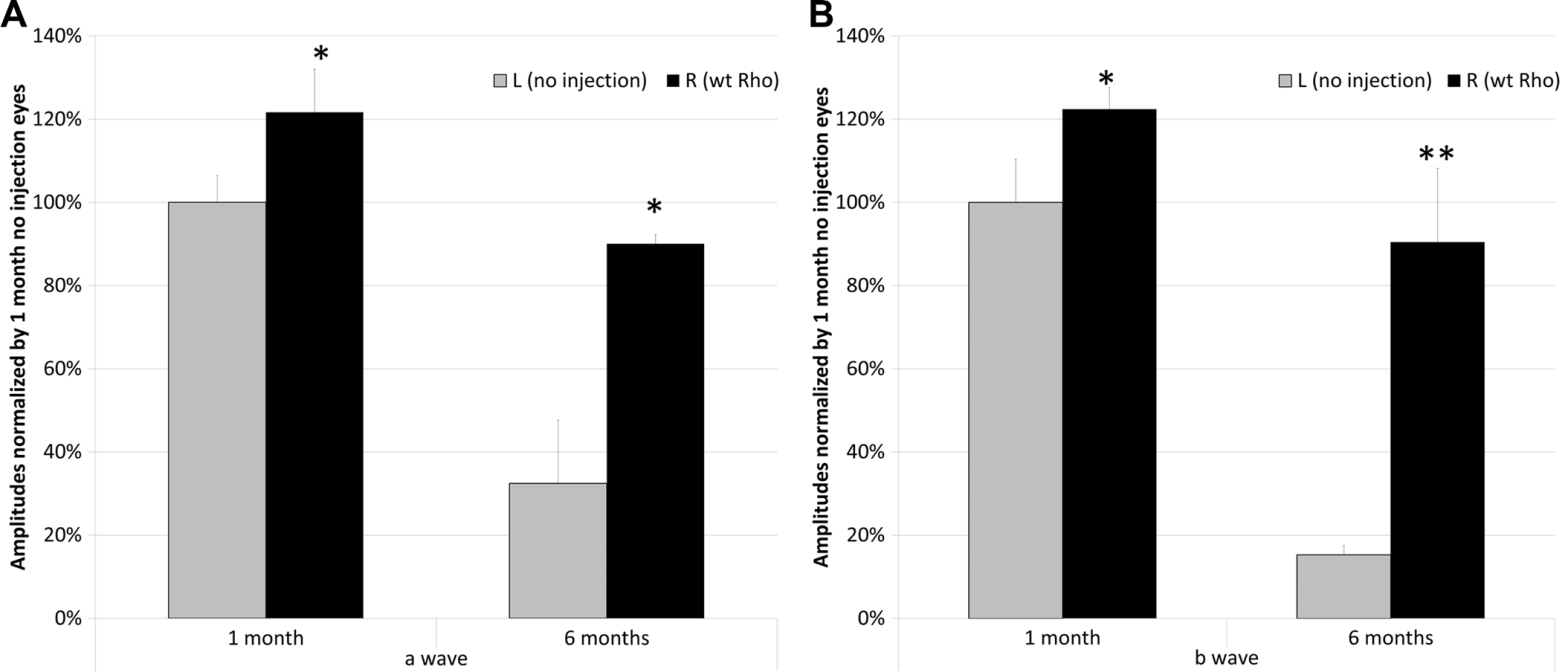
Improvement of electroretinography (ERG) response by single AAV injection of normal mouse rhodopsin cDNA (WT *Rho*) in P23H transgenic mice [89]. Bars represent the average of five scotopic ERG scans at 0 dB (2.6 cd (cd)-s/m^2^) a-wave response. **A**: and b-wave response. **B**: at 1 month and 6 months post injection. ERG amplitudes of 1 month uninjected P23H eyes were set as 100%. **A**: Compared with that of corresponding contralateral eyes, injection of AAV-*Rho* demonstrated a significant increase in a-wave amplitudes at both 1 month (122%) and 6 months (90%) time points. (*p<0.05, n=6). **B**: Compared with contralateral eyes, injection of WT *Rho* demonstrated the same significant increase in b-wave amplitudes as that of a-wave response at both 1 month (122%) and 6 months (90%) time points. (*p<0.05, ** p<0.005, n=6). Although injection injury can induce protective cytokines such as CNTF, this effect peaks within a few days of injection and is complete before the first measurements were made.

Although neurotrophic factors are aimed at preserving the photoreceptors without addressing the underlying mutation, more direct treatments including gene suppression or replacement are also being tested. Photoreceptors are sensitive to changes in rhodopsin levels, and overabundance of this protein can lead to retinal degeneration in mouse models [23,85]. In a transgenic mouse line, an excess of rhodopsin of only 23% was enough to cause RP-like retinal degeneration [86]. However, heterozygous null mutations in the mouse *Rho* gene result in a relatively normal retina at birth, with the length of rod outer segments approximately 60% that of *Rho+/+* mice [87]. Similar to humans bearing heterozygous null mutations, *Rho+/−* show a reduced response to light flash. Rhodopsin mutations, acting in a dominant negative manner, may be treatable simply through a DNA cassette expressing wild-type *RHO* [88,89]. With a single injection of AAV-transferred normal rhodopsin, 90% of the ERG response in a- and b-wave amplitudes can be preserved in P23H mice compared with P23H mice without treatment (Figure 2) [89]. Other mutations, however, may require suppression of the mutant gene plus provision of a wild-type replacement gene, especially since overexpression of rhodopsin can be toxic [86]. The methods for suppressing endogenous expression begin at the either the RNA or DNA level. RNA interference or ribozymes achieve suppression at the RNA level while advances in zinc finger transcription factors and endonucleases and transcription activator-like (TAL) effector nucleases could correct the mutations at the DNA level.

### Short interfering RNA

**Figure 3:**
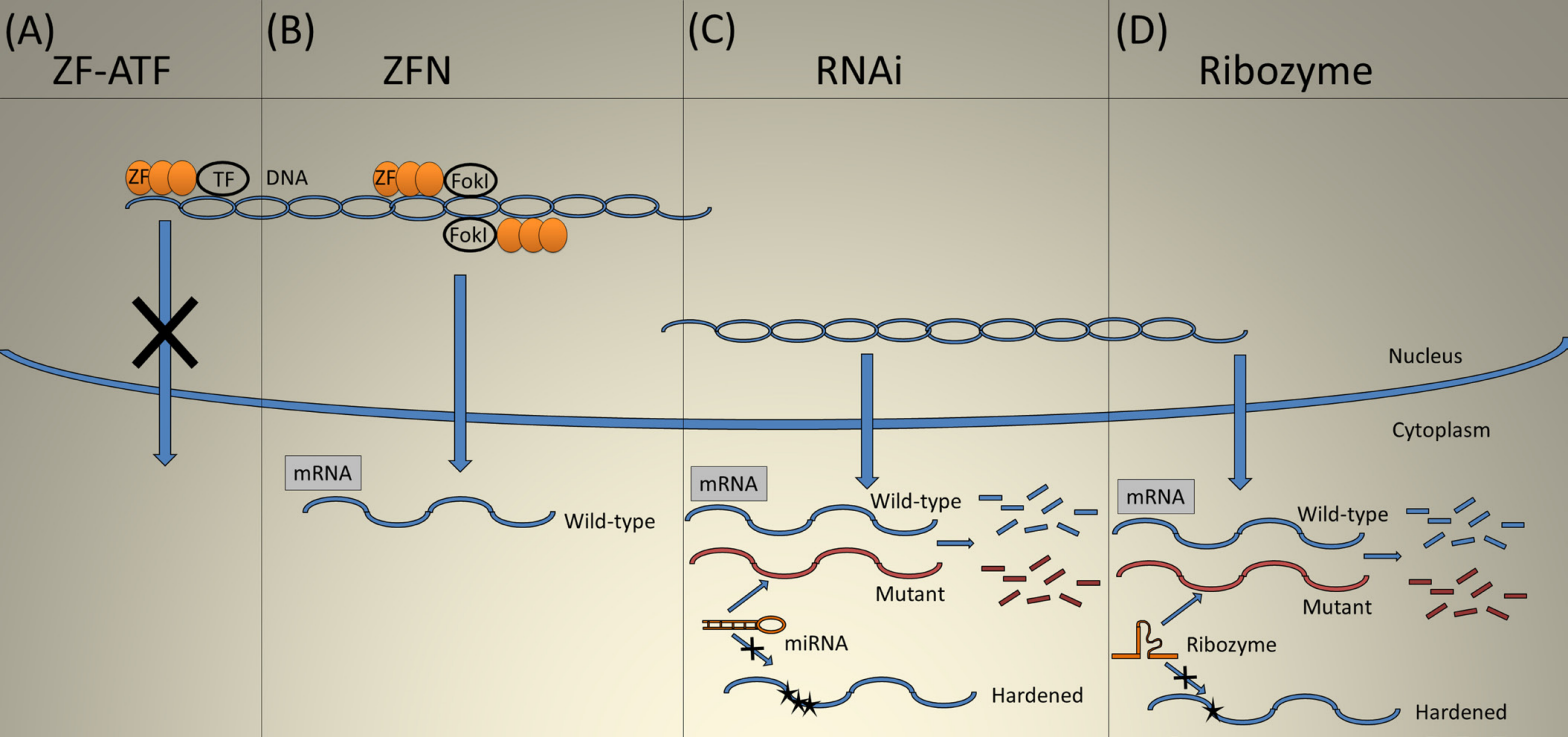
Gene suppression. **A**: Zinc finger artificial transcription factors (ZF-ATF) use suppressor transcription factors to silence gene transcription. **B**: Zinc finger nucleases (ZFN) causes a double-stranded break leading to correction of the mutation through recombination. **C**: miRNA and shRNA degrade the endogenous target transcript while sparing the introduced resistant mRNA (hardened) with an altered sequence. **D**: Ribozymes catalytically cleave the target transcript, but insertion of a guanosine at the target site dramatically reduces cleavage of the hardened target.

Therapeutic RNA interference (RNAi) employs three types of small RNA molecules: microRNA (miRNA), short hairpin RNA (shRNA), and short interfering RNA (siRNA). RNA interference with siRNA usually takes the form of direct treatment with modified double-stranded RNA (dsRNA) molecules, and requires repeated administration [90]. Therefore, this approach is not optimal for treating a chronic genetic disease, such as ADRP, though siRNAs are in clinical trials for age-related macular degeneration. Several groups are developing the use of RNAi for therapy for ADRP [91-97]. RNA interference was shown effective at knocking down the mutant mRNA and aiding in replacement with the wild-type gene [91,98] (Figure 3C).

### Short hairpin RNA

Stably expressed shRNAs can be used to selectively suppress expression of mutant *RHO* if there is sufficient nucleotide difference with the wild-type gene. A more generally applicable approach, however, is to use RNAi to suppress the production endogenous rhodopsin and allow expression of a sequence-altered *RHO* gene that is resistant to the shRNA [97-99]. Millington-Ward and colleagues used a two-vector approach to treat a mouse model of ADRP: one virus to deliver the shRNA and one to deliver the replacement rhodopsin cRNA. They reported retention of an average b-wave response of 60 microvolts at 20 weeks post injection in treated eyes, compared to a baseline response in control treated eyes. We have used a single AAV delivery vector to deliver a *RHO*-specific shRNA under the control of the H1 promoter and a resistant *Rho* gene under the control of the mouse opsin proximal promoter. We observed sustained protection of the retina (80% of the normal ERG response) up to 9 months post treatment with the combination vector [46].

### MicroRNA

The limitations of shRNAs and siRNA have led to the utilization of artificial miRNAs, which are derived from a natural pre-miRNA backbone such as miR-30 or miR-155 (101–106). Artificial miRNAs, avoid toxicity even when longer dsRNA regions are employed [100-102]. The natural antisense sequence, found in the miRNA, is removed and replaced with the siRNA sequence of interest [103]. Several structural properties are also necessary for potency: these include appropriate 3′ and 5′ flanking regions and a mismatch bulge at positions +1 and +11–12 [104]. Unlike shRNAs, artificial miRNAs allow for expression from Pol II promoters and from within natural or artificial introns [105,106]. This allows for greater temporal and spatial expression and incorporation of the artificial miRNA into an mRNA coding sequence. Multiple artificial miRNAs strung together may target several genes or multiple sites of the same gene [106,107]. Tested in cell and mouse lines, artificial miRNAs had fewer toxic effects and greater knockdown even when encoding the same siRNA as an equivalent shRNA when both were expressed from the U6 promoter [108-111]. Their effectiveness has also been studied in gene therapy and several disease models including HIV and cancer [111-114]. Mueller and colleagues recently demonstrated long-term prevention of the liver disease associated with a dominant mutation of α-1-antitrypsin using AAV delivery of a combination of concatenated artificial miRNAs and a resistant AAT cDNA [115].

### RNA interference disadvantages

Overexpressed shRNAs have been shown to be toxic in the central nervous system and the liver [116]. This is possibly due to the saturation of exportin 5 and obstruction of the normal miRNA maturation process. These instances of shRNA-associated toxicity required a duplex region of greater than 20 bp, and shorter duplexes appear to be safe [117]. Ambati and colleagues have reported that administering siRNAs greater than 21 bp can actually lead to retinal degeneration in animal models, through Toll-like receptor-3 (TLR3), but TLR7 and TLR8 are more commonly accepted as mediating the innate immune response to dsRNA [118,119]. Other potential problems of shRNAs and artificial miRNAs arise from “off target” effects [100-102]. These can arise from base pairing to the coding region or within the 3′ UTR of an unintended target mRNA. Fifteen consecutive base pairs can stimulate the RNAi response (mRNA digestion), but as few as seven base pairs in the 3′ UTR can inhibit translation via the miRNA pathway [120,121]. Double-stranded RNA such as siRNAs can also kindle non-specific antiviral defense mechanisms, leading to cell death [122].

### Ribozymes

Ribozyme technology for gene therapy arose in the early 1990s but has not advanced as rapidly as RNAi, and no commercial ribozyme has been marketed despite considerable effort. Nevertheless, small ribozymes such as hammerheads and hairpins are relatively easy to engineer and can be effective RNA knockdown agents in cells and in tissues (Figure 3D). The cleavage site for a hammerhead ribozyme has only moderate requirements, targeting NUX, where N is any nucleotide and X is any base except G [123-125]. There are differences in activity based on the target triplet, with AUC being the best followed by GUC and UUC [123-125]. Specificity is typically achieved through 12–15 base pairs with the target RNA [123,124]. The target for a hairpin ribozyme is BNGUC, where B is any nucleotide but adenosine. Because base pairing of the ribozyme is not assisted by a protein complex, experimental verification of the availability of targets within the folded RNA is important [126,127]. Since base pairing for either hammerheads or hairpins occurs in two distinct regions, there are no long contiguous regions of dsRNA in either ribozyme, minimizing their potential to stimulate the interferon pathway, though cleavage of an unintended RNA is still a problem. Regions of base pairing with the target mRNA are short, and it is relatively easy to use silent base changes to produce a cleavage-resistant mRNA: one mismatch is sometimes sufficient. The potential of ribozymes in treating ADRP has already been demonstrated in tissue culture and in animal models [91,92,96,128-130]. Ribozyme delivery with AAV led to reduced cleavage of a “hardened” (i.e., resistant) *RHO* transgene, preservation of ERG a- and b-wave amplitudes, and preservation of retinal structure based on histology.

### Zinc finger transcription factors

Zinc fingers (ZFs) are DNA-binding domains present in many transcription factors. A single zinc finger domain is composed of 30 amino acids forming a ββα fold and recognizes a three to four base pair target [131,132]. The targets can overlap at the fourth position, as the fourth base is bound on the opposite DNA strand. To ensure specificity, at least 16–18 base pairs or six ZF modules are needed [132,133]. The zinc finger modules can then be linked to transcriptional regulatory domains, typically repressors, or to endonucleases.

ZFs linked to transcriptional regulators are termed zinc finger artificial transcription factors (ZF-ATFs; Figure 3A). With repressor domains, ZF-ATFs have been effective at suppressing endogenous rhodopsin expression from wild-type and mutant *Rho* in a P347S transgenic mouse model of ADRP [134]. Mussolino et al. used a Krüppel-associated box repressor domain to silence both alleles (mutant and wild-type) of *Rho* [135]. Although this method reduced the rate of photoreceptor loss, this technique should be coupled to a gene replacement for full therapeutic results. In this case, base changes in the promoter driving expression of *RHO* could be introduced to prevent recognition by the ZF-repressor. A convenient way to do this would be to use the *RHO* promoter from another species, e.g., mouse promoter driving human *RHO*.

### Zinc finger nucleases

Zinc finger nucleases (ZFNs) provide a powerful tool capable of permanently and specifically altering the genomic DNA (Figure 3B). They are constructed using the DNA binding domains of two zinc finger modules dimerizing two subunits of the endonuclease domain of the nuclease FokI (or similar nuclease that cleaves downstream of its recognition domain) [133]. Each ZFN has a target sequence of about nine base pairs for a total recognition sequence of 18 bp. Cleavage of the target gene can lead to non-homologous end-joining to repair the chromosome or can lead to correction of the mutated sequence either through homologous recombination with the sister chromosome or through recombination with an ectopically administered DNA fragment with the corrected (wild-type) sequence. Recombination was increased to 17% using ZFN in a human P23H *RHO* expressing cell line [136].

### Transcription activator-like effector nucleases

Transcription activator-like effector nucleases, or TALENs, are reportedly easier to engineer than ZFNs and may supplant them in the molecular toolkit of gene therapists [137-139]. TALENs are produced by bacteria of the genus *Xanthomonas.* These proteins contain as many as 30 tandem repeats of a 33- to 35-amino-acid-sequence motif, but a pair of residues in each repeat allows for single nucleotide specificity. As with ZFNs, engineered TALENs include a FokI endonuclease domain to introduce a double-strand break in the target gene. The use of TALENs in targeted gene suppression has already been demonstrated by Wood and et al. in *C. elegans* [140].

### Zinc finger and TALEN disadvantages

Before ZFNs or TALENs are used in humans, the potential side effects need to be addressed. First, ZFNs are not completely specific and can induce toxic nonspecific cleavage [141]. The lack of specificity is largely due to one of the ZFNs binding and forming a homodimer. To overcome the formation of homodimers in ZFNs, small amino acid changes have been made with complementary changes on the other ZFN [142]. These changes destabilize homodimers while not affecting heterodimers. Another research group has attached destabilizing moieties, such as ubiquitin and FKBP12 [143]. These adducts allow control of protein levels and stability through the use of protease inhibitors nor a small ligand that stabilizes FKBP12 [141,143]. TALENs exhibited neither toxicity or non-specific mutagenesis in yeast grown on glucose medium, though no test of genotoxicity has been reported in mammalian cells [138]. Optimization of these techniques as well as increased screening and redesigning of ZFNs or TALENS before clinical use will likely provide a powerful tool for future retinal gene therapy [131,133].

### Additional pathways

Additional pathways to increase retinal survival include modification of the mammalian target of rapamycin (mTOR) pathway and delivery of molecular chaperones. The mTOR is a protein kinase regulated by various upstream signaling pathways. The level mTOR is upregulated in cones in some RP models, suggesting starvation for amino acids and/or glucose [144,145]. Administration of insulin to *rd1* mice led to a 50% increase in survival of cone photoreceptors [144].

Grp78 or Bip is an ER resident chaperone of the Hsp70 family. Although increased production of Bip is taken as a sign of ER stress, AAV delivery of Bip relieved ER stress and protected photoreceptors in a P23H *RHO* transgenic model of ADRP [32]. Misfolded rhodopsin can also be cleared from the endoplasmic reticulum by the so-called ERAD pathway and VCP, a molecular chaperone involved in that pathway, may have therapeutic potential for treating ADRP [146]. As an alternative to gene therapy, pharmacologic chaperones have great as treatment for diseases such as ADRP that are associated with misfolded proteins [12,147].

### Therapeutic delivery

#### Viral mediated delivery

ADRP gene therapy has relied heavily on viral mediated delivery [148]. Lentivirus, adenovirus and AAV vectors have proven effective at delivering genes to the retina and RPE for ocular gene therapy [149]. Lentivirus delivers genes efficiently to the RPE, but AAV is probably the most versatile vector due to its wide range of host cells, DNA-based genome, no known pathogenicity, and many serotypes with differing tissue specificity [6,150-152]. Several methods have already been created for large-scale production of recombinant AAV (rAAV), and it is already in clinical trials for Leber congenital amaurosis (LCA) associated with mutations in RPE65 [153-158]. Due to these advantages, AAV has been used to develop treatments for many forms of retinal degeneration, including achromatopsia, retinoschisis, X-linked RP, recessive RP, and dominant RP [6,49,159-167]. AAV, however, can accommodate only a 4.7 kb insert. This limitation does not preclude most ADRP genes, neurotrophic factors, or small shRNA and miRNA [6]. Recombination between rAAV genomes in cells infected with two viruses can increase the delivery capacity of AAV, albeit at reduced efficiency [168]. For larger genes and promoters, lentiviral vectors can be used, as they offer the greater carrying capacity at 8 kb [1,148,169,170]. Helper-dependent adenoviral vectors have little remaining genetic material between the terminal repeats and therefore have a high capacity for inserts (about 35 kb) [48]. Vector production requires helper viruses that cannot be completely removed, so safety may be an issue. Another significant problem is that neither lentiviral vectors nor adenoviral vectors transduce photoreceptors efficiently, thus limiting the vectors’ utility for treating ADRP. Modifying the lentiviral pseudotype has not improved photoreceptor transduction substantially [171,172]. Deletion of the RGD sequence in the penton base of Ad5 improved infection of photoreceptors, but transduction efficiency lags behind that of AAV5 or AAV8 [173]. A recently developed “gutted” version of Sendai virus has been used for RPE transduction in mice and rats, and this RNA virus may be particularly useful for rapid onset delivery of siRNA [174].

### Disadvantages of viral delivery

Despite the clear advantages to viral vectors, all viruses have size limitations, differing degrees of immune response mediated by Müller cell activation, and inflammation [149,170,175]. The humoral response to viral infection can prevent readministration of the same virus serotype following intravitreal injection [176]. In addition, neutralizing antibodies to AAV resulting from current or previous infections may attenuate gene transfer and should preclude participation in a clinical trial. Due to the size of the particles and the blood–retinal barrier, ocular injections are used for retinal gene transfer. However, in neonatal mice systemic injection of AAV9 transduces the retina, though systemic injections are likely to elicit an immune response and to transfer genes to the spleen, lung, and liver. For these reasons, alternatives to viral vectors are being investigated [177].

### Non-viral delivery

Gene transfer to photoreceptors with liposomes has been inefficient in animal models. In contrast, DNA delivery using several types of nanoparticles has had more success. Nanoparticles may circumvent many of the disadvantages to viral vectors including limited carrying capacity and the immunogenic response to viral capsid proteins. Gene and siRNA delivery has been shown in intestinal cells with the use of orally administered nanoparticles [178]. Additional success with nanoparticles has been demonstrated in the lungs and central nervous system [1,179]. Delivery of the nanoparticles may be aided by gene gun or electrotransfer, which have proven useful for ciliary muscles and the cornea; however, the gene gun is not suitable for gene transfer to the retina [149]. Electroporation produced lasting expression of GDNF and GFP in the retina [149,180]. Additionally, electroporation has been effective in cultured retinal cells and retinal explants, and electroporation has been used in animals for plasmid and siRNA delivery to the retina [93,181,182].

CK30-PEG nanoparticles have shown promise in mouse models of ADRP [183,184]. CK30-PEG nanoparticles are composed of poly-L-lysine coated with polyethylene glycol (PEG) moieties [183,184]. Together, these neutralize the negative charge of DNA, allowing for DNA compaction. PEG groups prolong circulation by preventing protein attachment and slow DNA degradation [183]. The carrying capacity has been tested up to 20 kb, four times larger than AAV, and provided robust gene expression [183]. Moreover, the gene activation was within hours, while AAV requires a minimum of 2 days. The size is only 8–20 nm, allowing passage through the tight junctions in the retina [183]. Injections of nanoparticles containing wild-type rds/peripherin (*Perph2*) in *Perph2^+/−^* mice at postnatal day 5 mice led to protection of the retina as demonstrated by IRBP and opsin expression out to 15 months [183]. Using either the IRBP promoter or the chicken beta actin promoter to drive rds/peripherin expression, *Perph2^+/−^* mice showed increased ERG response and improved retinal structure [185]. Additional studies measured onset of luciferase and LacZ expression following retina and corneal nanoparticle delivery [186]. CK30-PEG showed no signs of an immunogenic response or of toxicity [1,187].

### Disadvantages of non-viral delivery

Currently, nanoparticles have several disadvantages. Nanoparticles are typically degraded quickly in endosomes, and delivered genes may have a shorter duration of expression than viral vectors [186]. Long-term gene delivery has been reported using CK30-PEG nanoparticles [183-185], but these results must be replicated in other laboratories. Because the outer retina is not well perfused, nanoparticles may have a long residence time, and formulations containing gold or iron that may be suitable for gene delivery in larger organs may prove toxic in the retina [188]. In addition, some nanoparticles may induce light damage to the retina [189].

### Future directions

The future of ocular gene therapy may not lie in any one treatment discussed above but in the combination of treatments. NTFs are effective at retarding photoreceptor loss, but the degeneration is only slowed, and a more permanent gene-directed treatment may be needed. NTFs could be used as an initial treatment to protect and slow photoreceptor loss. This approach would prove most useful in rapidly degenerating forms of ADRP. Second, a combination of ribozymes, miRNA, and/or shRNAs could be used to ensure sufficient suppression of the mutant allele. Third, advancements in ZFNs or TALENs to reduce toxic off-site cleavage will make these powerful tools for permanently correcting the mutant allele at the DNA level. This technology partnered with stem cell–based regenerative medicine may permit repair of a patient’s own stem cells for replacing defective photoreceptors or RPE.

The use of self-complementary AAV and capsid modifications will improve AAV-mediated gene delivery [161,190,191]. Gene therapy using self-complementary AAV permits delivery of double-stranded DNA to shorten the onset of gene expression and increases transduction efficiency [192-197]. Capsid modification of AAV has also yielded improvements in transduction [177,190,198,199]. Because phosphorylation of surface tyrosine residues leads to degradation of AAV particles after infection, mutating certain tyrosines to phenylalanine increases productive infection. Although AAV serotypes 5 and 8 have been the vectors of choice for photoreceptors, the modified AAV8 (Y733F) has increased transduction efficiency [6,190,191,200].

Finally, nanoparticles hold a great potential for treating not just the eyes but also the entire central nervous system, as these particles have the ability to pass through the blood/central nervous system barriers, have low immunogenicity, and allow for large insert sizes. Although further research is needed, advances in nanoparticles may allow for topical or systemically delivered gene therapies to the retina.
